# Supplementary material for: Cortical parvalbumin neurons are responsible for homeostatic sleep rebound through CaMKII activation
Source: Nat Commun. 2024 Jul 18;15:6054. doi: 10.1038/s41467-024-50168-5 (PMC11258272; doi:10.1038/s41467-024-50168-5)
Supplement: Supplementary file 1 — Supplementary Information [file 41467_2024_50168_MOESM1_ESM.pdf]

Nature Communications

Supplementary information

Cortical parvalbumin neurons are responsible for homeostatic sleep rebound through CaMKII activation

Kazuhiro Kon, Koji L. Ode, Tomoyuki Mano, Hiroshi Fujishima, Riina R. Takahashi, Daisuke Tone, Chika Shimizu, Shinnosuke Shiono, Saori Yada, Kyoko Matsuzawa, Shota Y. Yoshida, Junko Yoshida Garçon, Mari Kaneko, Yuta Shinohara, Rikuhiro G. Yamada, Shoi Shi, Kazunari Miyamichi, Kenta Sumiyama, Hiroshi Kiyonari, Etsuo A. Susaki, and Hiroki R. Ueda

Supplementary Fig. 1–14

Supplementary Table 1

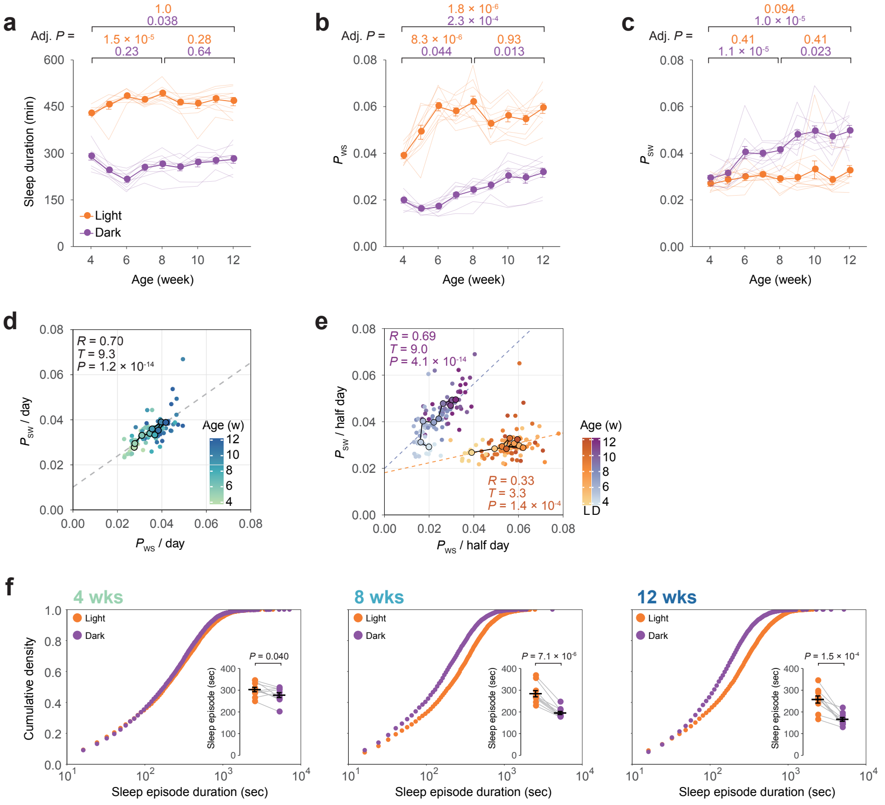

Supplementary Fig. 1

Supplementary Fig. 1: Analysis of developmental changes in sleep architecture.

(a–c) Half-day sleep duration **(a)**, P_{ws} **(b)**, and P_{sw} **(c)** in the developing mice (n = 10). At each age, the orange and purple points represent the mean sleep parameters during the light phase (first half of the day) and the dark phase (second half of the day). Two-sided Welch's t-test with Bonferroni correction was used on animals aged 4, 8, and 12 weeks.

(d, e) Correlation diagram of P_{WS} and P_{SW} in developing mice over a day **(d)** and half-day **(e)**. The mean of P_{WS} and P_{SW} at each age is shown by black-fringed dots, while other dots show the P_{WS} and P_{SW} of individual developing mice, whose color corresponds to their age. At each age, orange and purple points represent the parameters during the light phase and the dark phase, respectively **(e)**. The regression line is represented by each dashed line. The diagrams depict the correlation coefficient (R), T-, and P-value.

(f) Cumulative frequency plot of sleep episodes. The ages of 4, 8, and 12 weeks are chosen representative data. Two-sided Welch's t-test was used to compare the mean sleep episodes in light and dark phases at 4, 8, and 12 weeks old (n = 10, at each age).

Line plots show mean ± SEM. Source data are provided as a Source Data file.

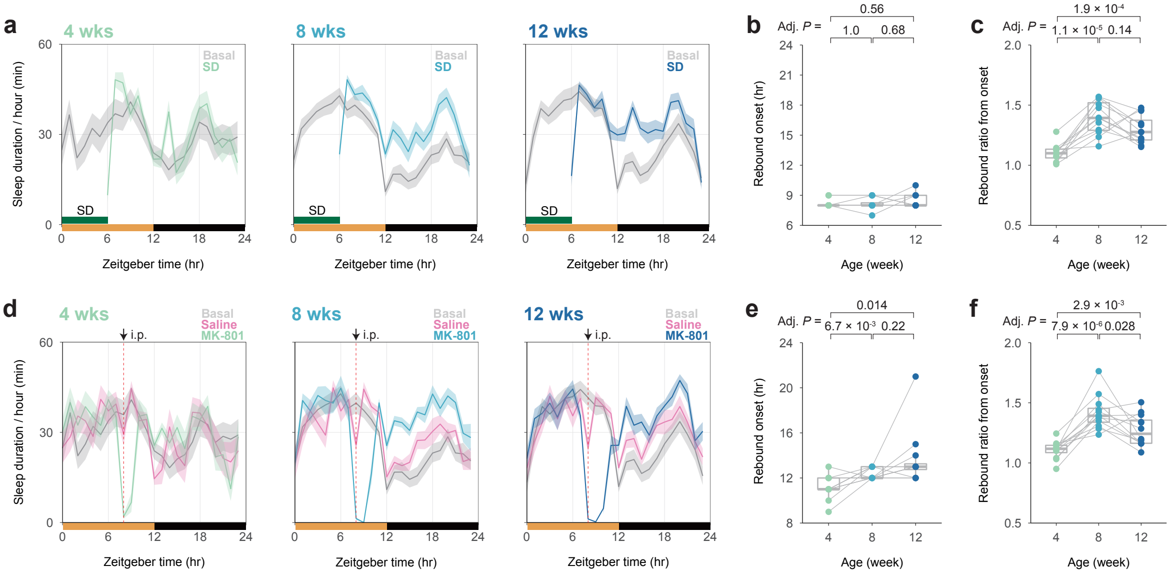

Supplementary Fig. 2

Supplementary Fig. 2: Analysis of developmental changes in sleep homeostasis.

(a, d) Hourly sleep duration over 24 hours in developing mice (n = 12) at 4, 8, and 12 weeks old during sleep deprivation **(a)** and MK-801 administration **(d)**. In the panels, the basal (gray line) is the same.

(b, e) Rebound onset is defined as the point when sleep duration after SD exceeds the corresponding value in the Basal **(b)** or when sleep duration after MK-801 administration exceeds the corresponding value in the day of saline administration **(e)**. Individual mouse dots are connected by gray lines. Between the ages, two-sided Welch's *t*-test with Bonferroni correction was used.

(c, f) Sleep rebound ratio in SD (SD / Basal) **(c)** or in MK-801 administration (MK-801 / Saline) **(f)** was computed from the rebound onset to ZT24 in individual mice. Individual mouse dots are connected by gray lines. Between the ages, two-sided Welch's *t*-test with Bonferroni correction was used.

Line plots show mean ± SEM. In box plot, boxes show the median, 25th and 75th percentiles, and whiskers show minima to maxima excluding outliers. Source data are provided as a Source Data file.

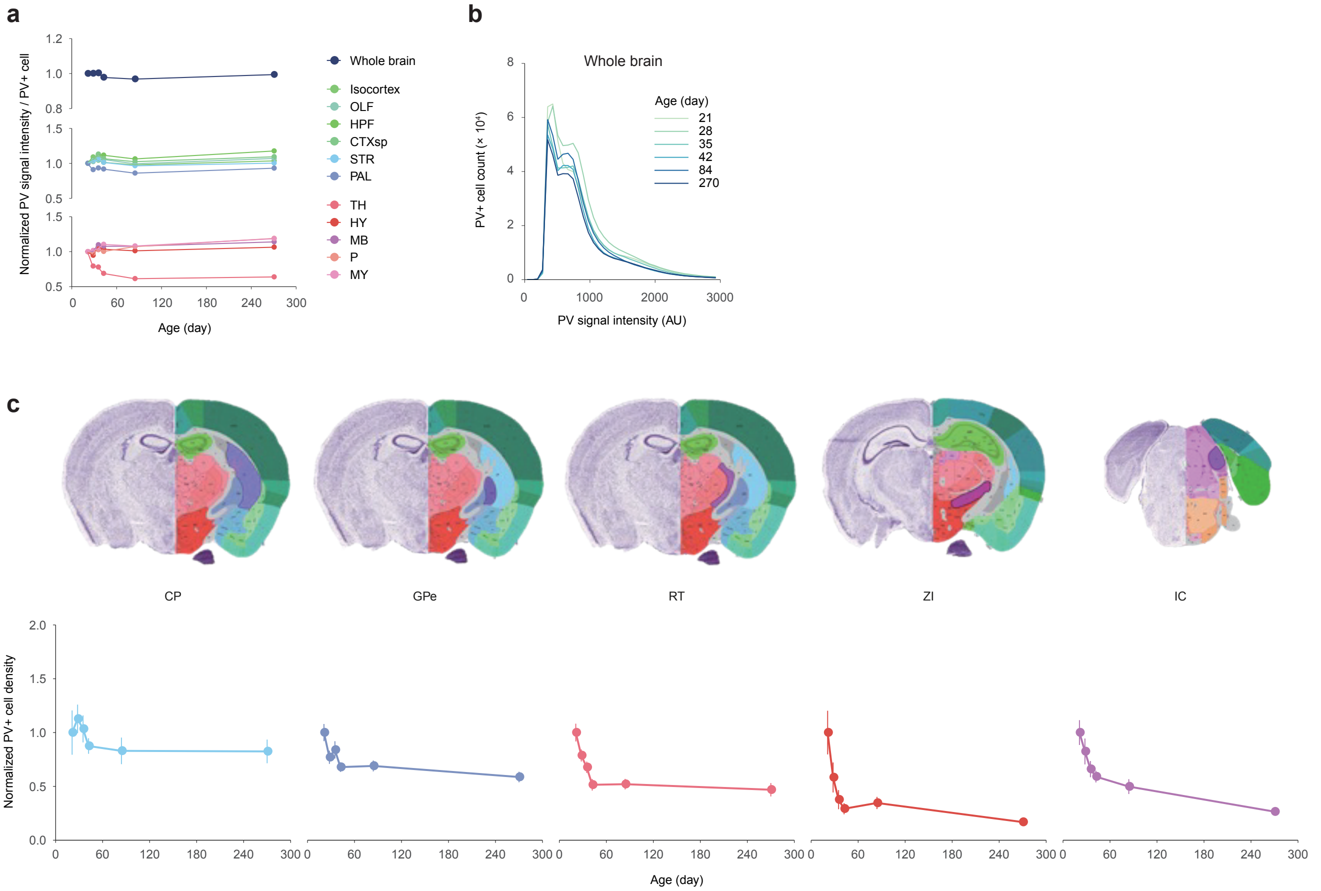

Supplementary Fig. 3

Supplementary Fig. 3: Whole-brain analysis of PV neurons across post-weaning development.

(a) The mean of PV signal intensity per PV+ cell in the whole brain (top), cerebral regions (middle), and brain-stem regions (bottom) across post-weaning development (n = 6 for each age). Each point represents the mean value of each age normalized by that of P21.

(b) The distribution of PV signal intensity per PV+ cell over the entire brain at each age.

(c) The mean of PV+ cell density in selected subcortical and brain-stem regions during post-weaning development (n = 6 for each age). Each point represents the mean value of each age normalized by that of P21, and the colors displayed correspond to the brain regions depicted in **(a)**. Images of coronal brain slice were from the Allen Brain Atlas.

Line plots show mean ± SEM. The Allen Brain Atlas ontology is used to define brain region acronyms. Source data are provided as a Source Data file.

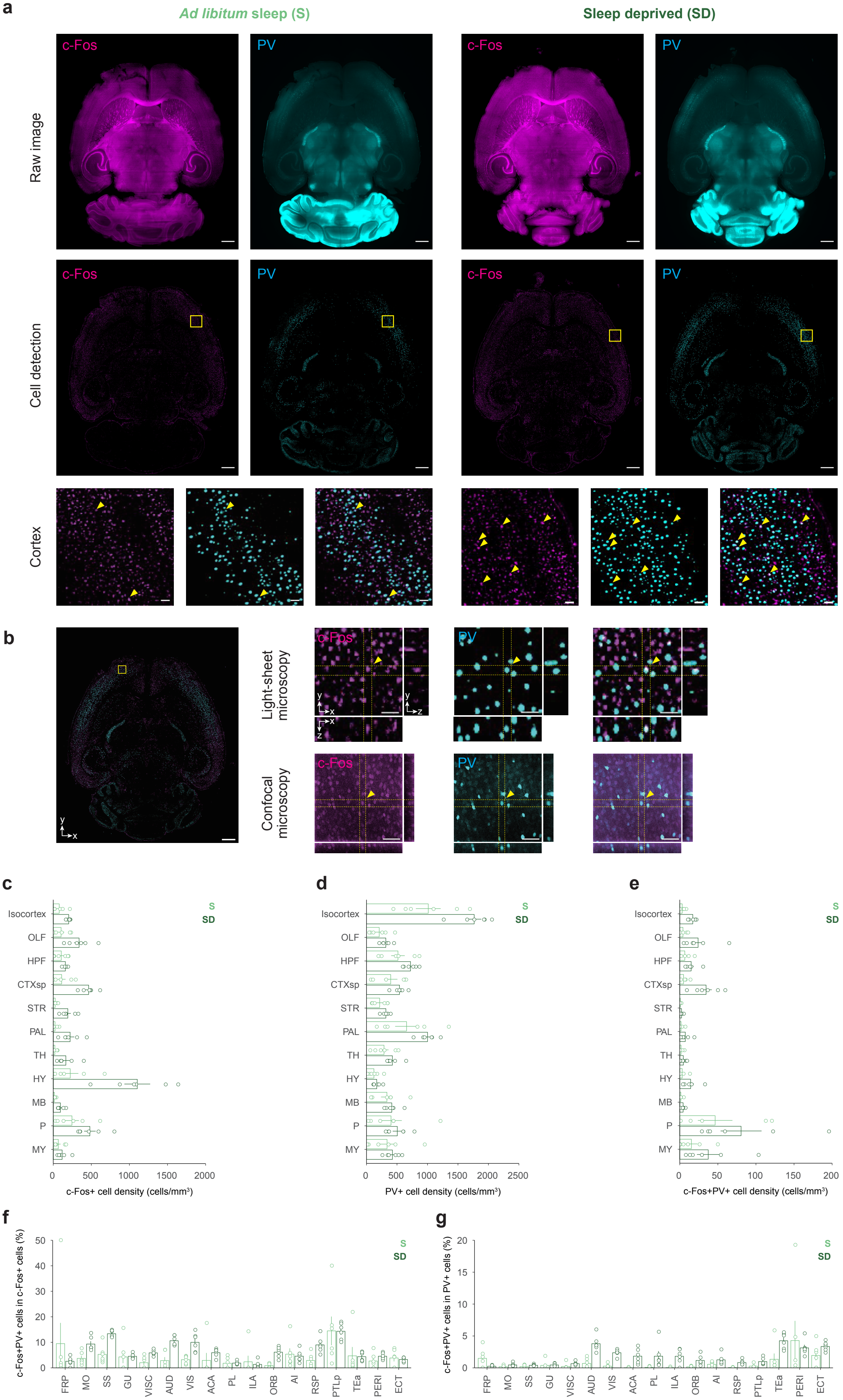

Supplementary Fig. 4

Supplementary Fig. 4: Analysis of cortical PV-neuron activity upon sleep deprivation.

(a) Representative images for c-Fos and PV immunostaining in control (S) and sleep-deprived (SD) brains. Raw images (top) and detected cell images in macro-views (middle) and zoomed-in views (bottom; yellow boxes in middle panels) are shown. Yellow arrowheads indicate the position of c-Fos+PV+ cells. Maximum intensity project spanning 35 μm thickness. Scale bars = 1 mm (top and middle) or 100 μm (bottom).

(b) Comparison between light-sheet and confocal images for c-Fos and PV immunostaining. Representative images obtained by light-sheet microscopy (top-right) and confocal microscopy (bottom-right) of the selected cortical region (indicated by yellow box in the left panel) are displayed. The images of coronal (z-x) and sagittal (y-z) sections are digitally reconstructed. Maximum intensity project spanning 35 μm thickness both in light-sheet microscopy and confocal microscopy. The position of one of the c-Fos+PV+ cells is indicated by yellow arrowheads and demarcated by yellow lines. Scale bars are indicated as 1 mm (left) or 100 μm (right).

(c–e) Cell density of c-Fos+ cells **(c)**, PV+ cells **(d)**, and c-Fos+PV+ cells **(e)** in major brain regions after SD (*n* = 6 for each group).

(f, g) Rate of double-positive (c-Fos+PV+) cells in c-Fos+ cells **(f)** or PV+ cells **(g)** in each region of the isocotex after SD (*n* = 6 for each group).

Bar plots show mean ± SEM. The Allen Brain Atlas ontology is used to define brain region acronyms. Source data are provided as a Source Data file.

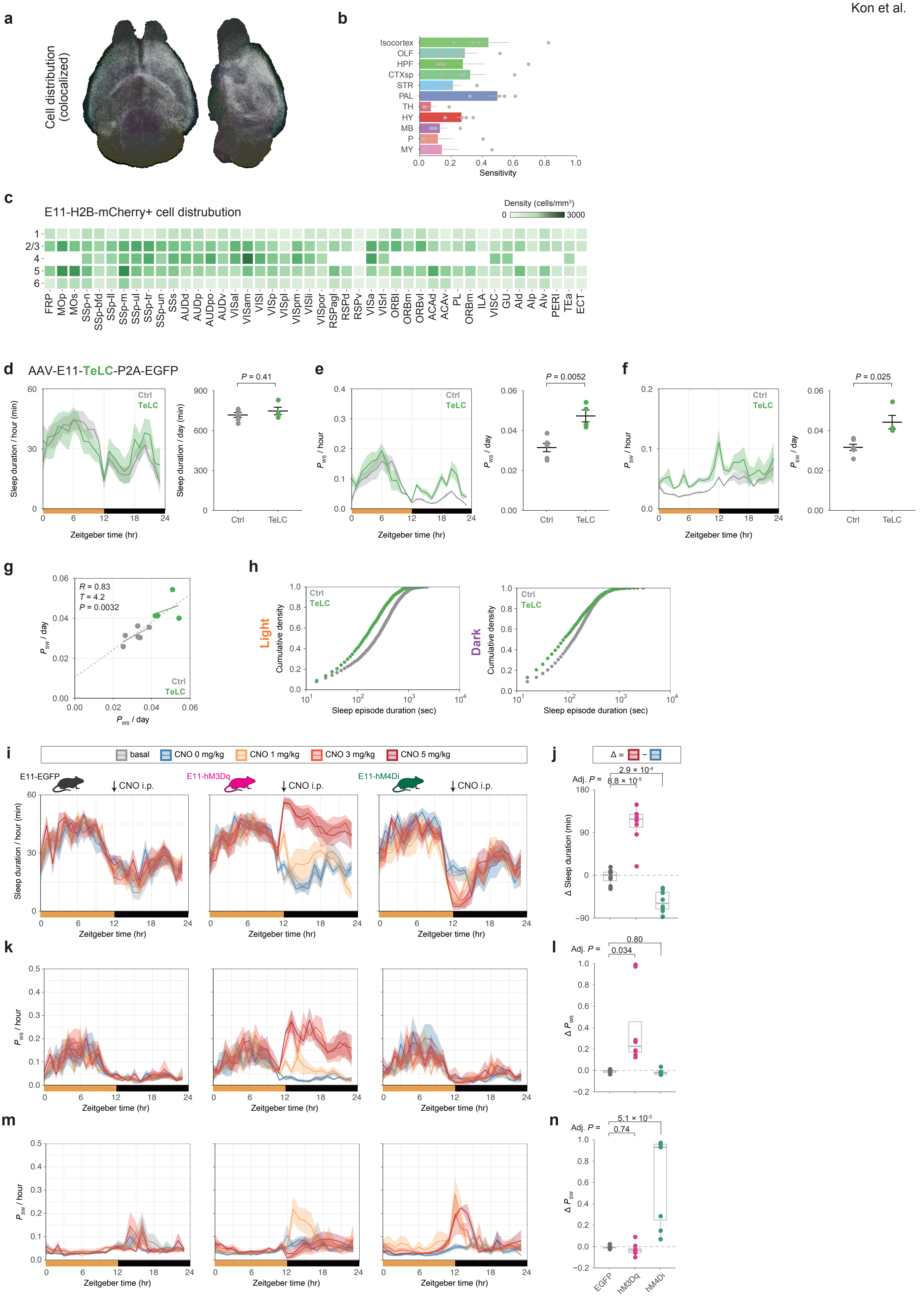

Supplementary Fig. 5

Supplementary Fig. 5: Chronic or acute manipulation of E11-neuron activity changes the sleep-wake states.

(a) Representative whole-brain views of dorsal and lateral colocalized (mCherry+PV+) cells, using the source brain shown in **Fig. 3j**.

(b) Sensitivity was calculated by dividing the number of colocalized (mCherry+PV+) cells by the total number of PV+ cells in the major brain regions.

(c) A region-wise heat map of E11 neuron density (mCherry+ cells) in the isocortex.

(d–f) Daily sleep duration **(d)**, P_{WS} **(e)**, and P_{SW} **(f)** in Ctrl (n = 6) and E11-TeLC (n = 4) mice. The groups were compared using two-sided Welch's t-test.

(g) Daily P_{WS} and P_{SW} correlation diagrams in the Ctrl (gray) and E11-TeLC (green) mice. The values represented are the same as those shown in the panels **(e)** and **(f)**. Each dot represents a single mouse. The regression lines for the Ctrl, E11-TeLC, and all data are represented by gray-solid, green-solid, and gray-dashed lines, respectively. The diagram depicts the correlation coefficient (R), T-, and P-value for all data.

(h) Cumulative frequency plot of sleep episodes in the Ctrl (gray) and E11-TeLC (green) mice during the light or dark phase.

(i, k, m) Hourly sleep duration **(i)**, P_{WS} **(k)**, and P_{SW} **(m)** over 24 hours on the basal day (the mean of two days with no injection) and days with CNO injection. Each mouse received i.p. injection in turn: 0, 1, 3, or 5 mg/kg CNO injection at ZT12 with at least 2 days between injections (n = 8 for each group).

(j, l, n) The differences (Δ) in sleep duration **(j)**, P_{WS} **(l)**, and P_{SW} **(n)** between the 0 and 5 mg/kg CNO conditions during ZT12–15. Each dot represents a single mouse. Two-sided Welch's t-test with Bonferroni correction was used to compare the groups to the E11-EGFP mouse group.

Line and interval plots show mean ± SEM. In box plot, boxes show the median, 25th and 75th percentiles, and whiskers show minima to maxima excluding outliers. Brain region acronyms follow the ontology defined by the Allen Brain Atlas. Source data are provided as a Source Data file.

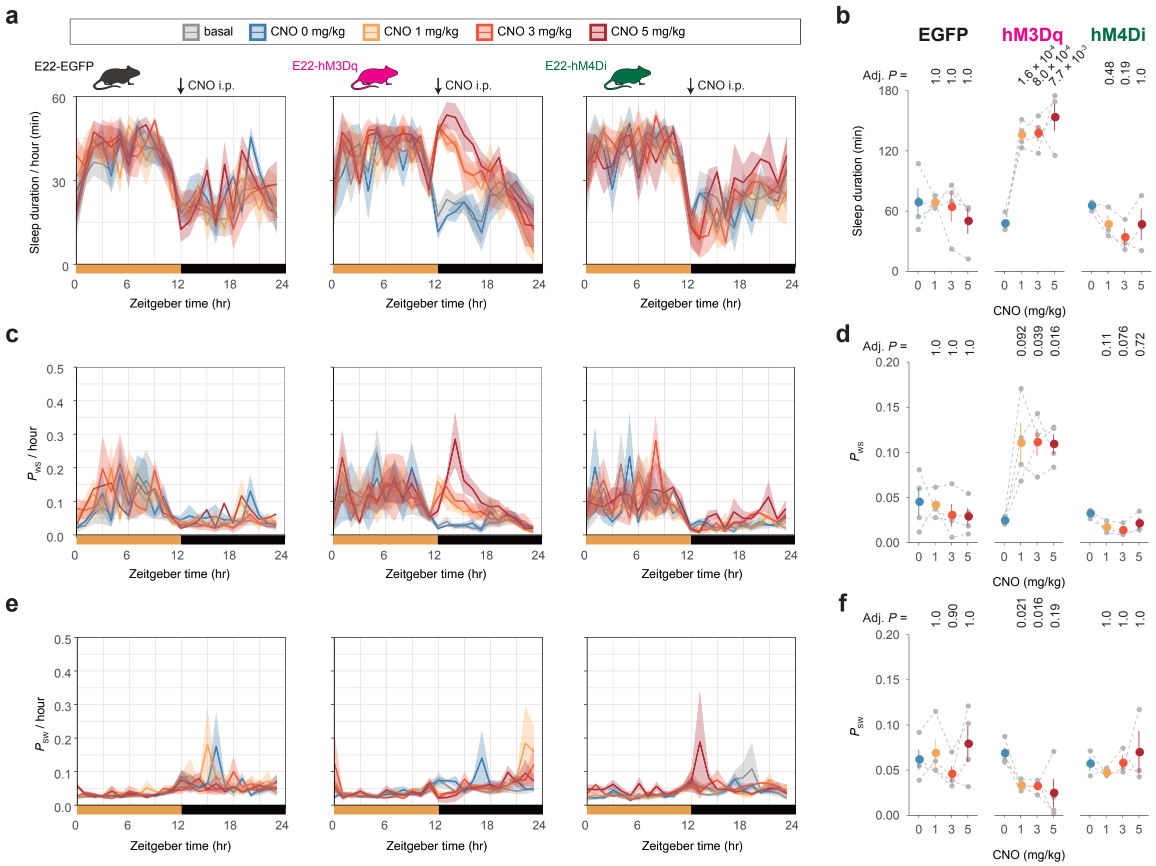

Supplementary Fig. 6

Supplementary Fig. 6: Acute manipulation of E22-neuron activity changes the sleep-wake states.

(a, c, e) Hourly sleep duration **(a)**, P_{WS} **(c)**, and P_{SW} **(e)** over 24 hours on the basal day (the mean of two days with no injection) and days with CNO injection. Each mouse received i.p. injection in turn: 0, 1, 3, or 5 mg/kg CNO injection at ZT12 with at least 2 days between injections (n = 4 for each group, except for E22-hM4Di (n = 3)).

(b, d, f) Sleep duration **(b)**, P_{WS} **(d)**, and P_{SW} **(f)** on the days with CNO injection during ZT12–15. Each gray dot represents an individual mouse, while colored dots represent the mean in each condition. Individual's mouse dots are connected by gray-dashed lines. Within the groups, two-sided Welch's *t*-test with Bonferroni correction were run against the 0 mg/kg CNO condition.

Line and interval plots show mean ± SEM. *P < 0.05, **P < 0.01, ***P < 0.001, n.s. = non significance. Source data are provided as a Source Data file.

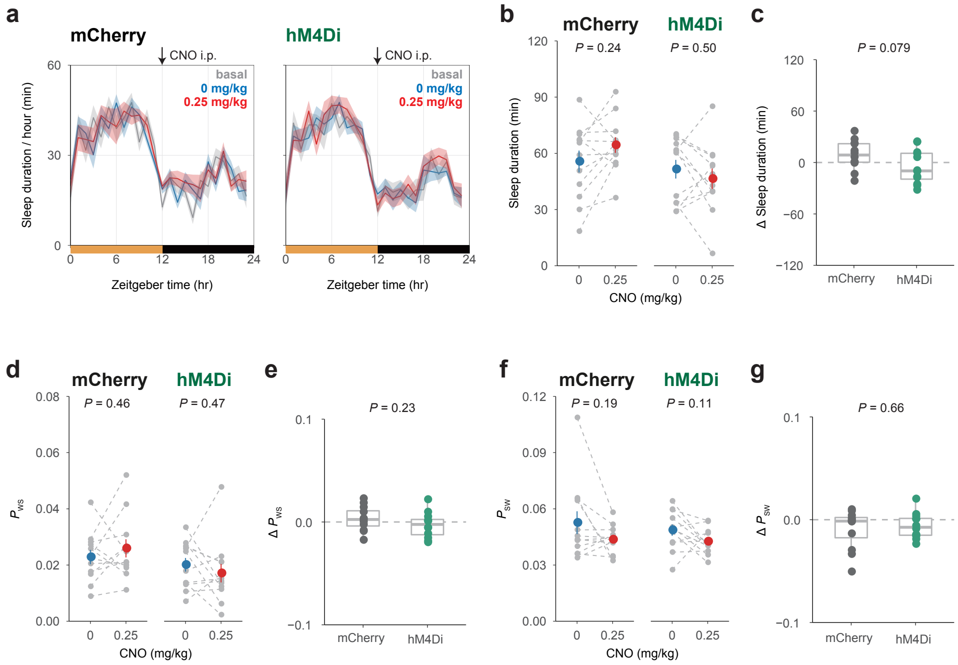

Supplementary Fig. 7

Supplementary Fig. 7: Suppression of E11 neurons by the low-dose CNO administration does not affect basal sleep architecture.

(a) Hourly sleep duration over 24 hours on the baseline day (gray; the mean of two days with no injection) and days with CNO injection. Each mouse received i.p. injection in turn: 0 (blue) or 0.25 (red) mg/kg CNO injection at ZT12, with at least 2 days between injections (n = 12 for each group).

(b, d, f) Sleep duration **(b)**, P_{WS} **(d)**, and P_{SW} **(f)** on the days with CNO injection during ZT12–15. Each gray dot represents an individual mouse, while the colored dots represent the mean in each condition. Individual mouse's dots are connected by gray-dashed lines. Two-sided Welch's t-test was used to compare conditions within groups.

(c, e, g) The differences (Δ) in sleep duration **(c)**, P_{WS} **(e)**, and P_{SW} **(g)** between days with 0 and 0.25 mg/kg CNO injections during ZT12–15. The group were compared using two-sided Welch's t-test.

Line and interval plots show mean ± SEM. In box plot, boxes show the median, 25th and 75th percentiles, and whiskers show minima to maxima excluding outliers. Source data are provided as a Source Data file.

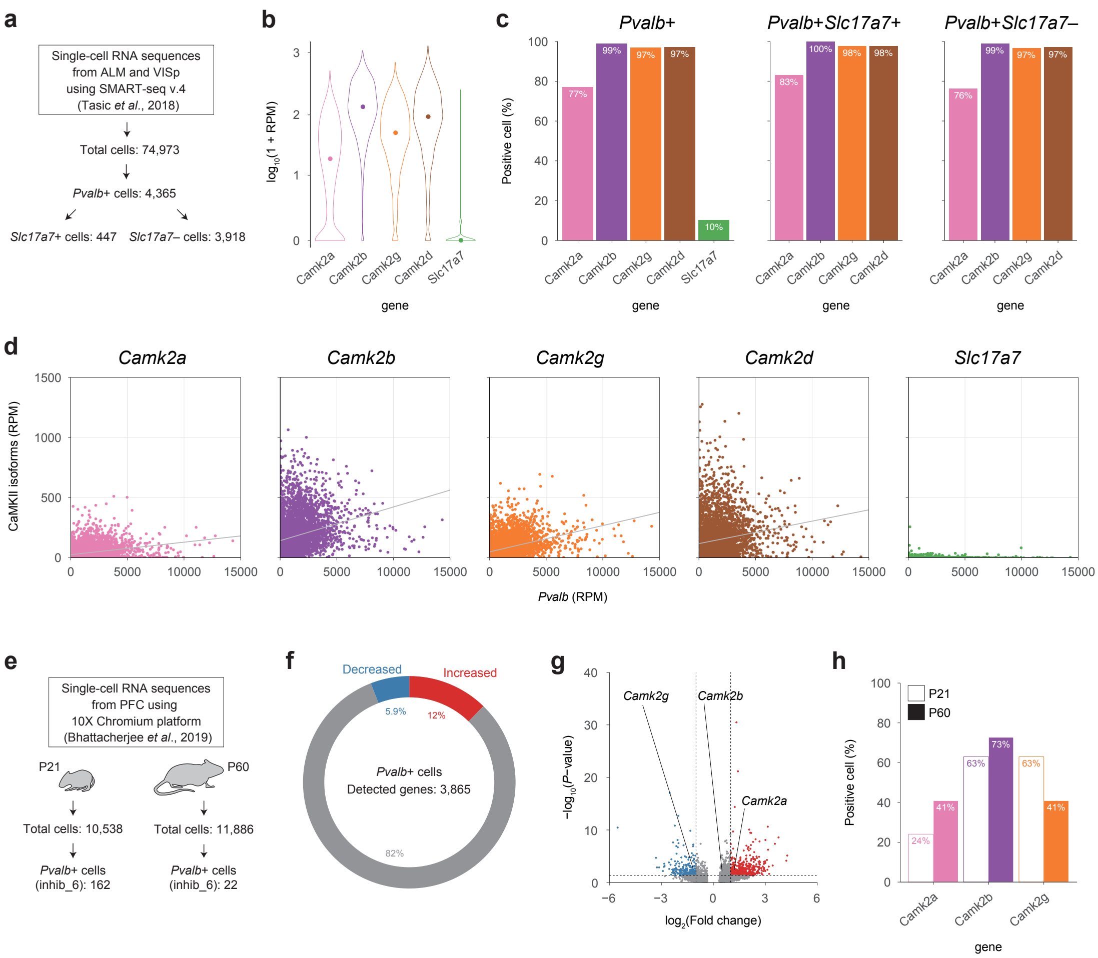

Supplementary Fig. 8

Supplementary Fig. 8: Single-cell RNA sequence analysis of CaMKII expression in cortical *Pvalb*+ cells.

(a) Single-cell RNA sequence analysis in the adult (P53–59) mouse cortex (ALM or VISp) using the Allen Brain Institute data[40]. *Pvalb*+ cells were the cells in the data that were assigned the *Pvalb* subclass. *Pvalb*+ cells were further subdivided into *Slc17a7*-positive (*Pvalb*+*Slc17a7*+) and negative (*Pvalb*+*Slc17a7*−) cells (*Slc17a7*: glutamatergic neuron marker gene). The cell numbers used for analysis in the panels **(b–d)** are shown.

(b) Violin plots depict gene expression of all CaMKII isoforms and *Slc17a7* in *Pvalb*+ cells (n = 4,365). On a $\log_{10}$ scale, expression values (reads per million, RPM) are displayed. The medians are indicated by colored dots.

(c) Positive cell (Reads > 0) rate in *Pvalb*+ (n = 4,365, left), *Pvalb*+*Slc17a7*+ (n = 447, center), and *Pvalb*+*Slc17a7*− (n = 3,918, right) cells.

(d) Correlation diagram of the *Pvalb* and each selected gene (CaMKII isoforms and *Slc17a7*) expression values (RPM) in *Pvalb*+ cells (n = 4,365). Each dot represents a single cell. The gray lines represent the regression lines.

(e) Method for comparing single-cell RNA sequences in the adolescent (P21) and young-adult (P60) mouse prefrontal cortex (PFC) using data from the Yi Zhang group[41]. The cells in the data assigned to the Inhib-6 cluster were dubbed *Pvalb*+ cells.

(f, g) Donut plot **(f)** and volcano plot **(g)** of up-regulated (red) or down-regulated (blue) gene expression (n = 3,865 genes) in *Pvalb*+ cells from P21 (n = 162 cells) to P60 (n = 22 cells) mouse PFC (cutoff: averaged fold change > 2 and P-value < 0.05). The volcano plot **(g)** highlights CaMKII isoform genes that have been identified.

(h) Positive cell (Reads > 0) rate of each CaMKII isoform gene in *Pvalb*+ cells of mouse PFC at P21 (n = 162 cells) and P60 (n = 22 cells). P21 and P60 are represented by edged and filled bars, respectively.

Abbreviations: ALM, the anterior lateral motor cortex; VISp, the primary visual cortex.

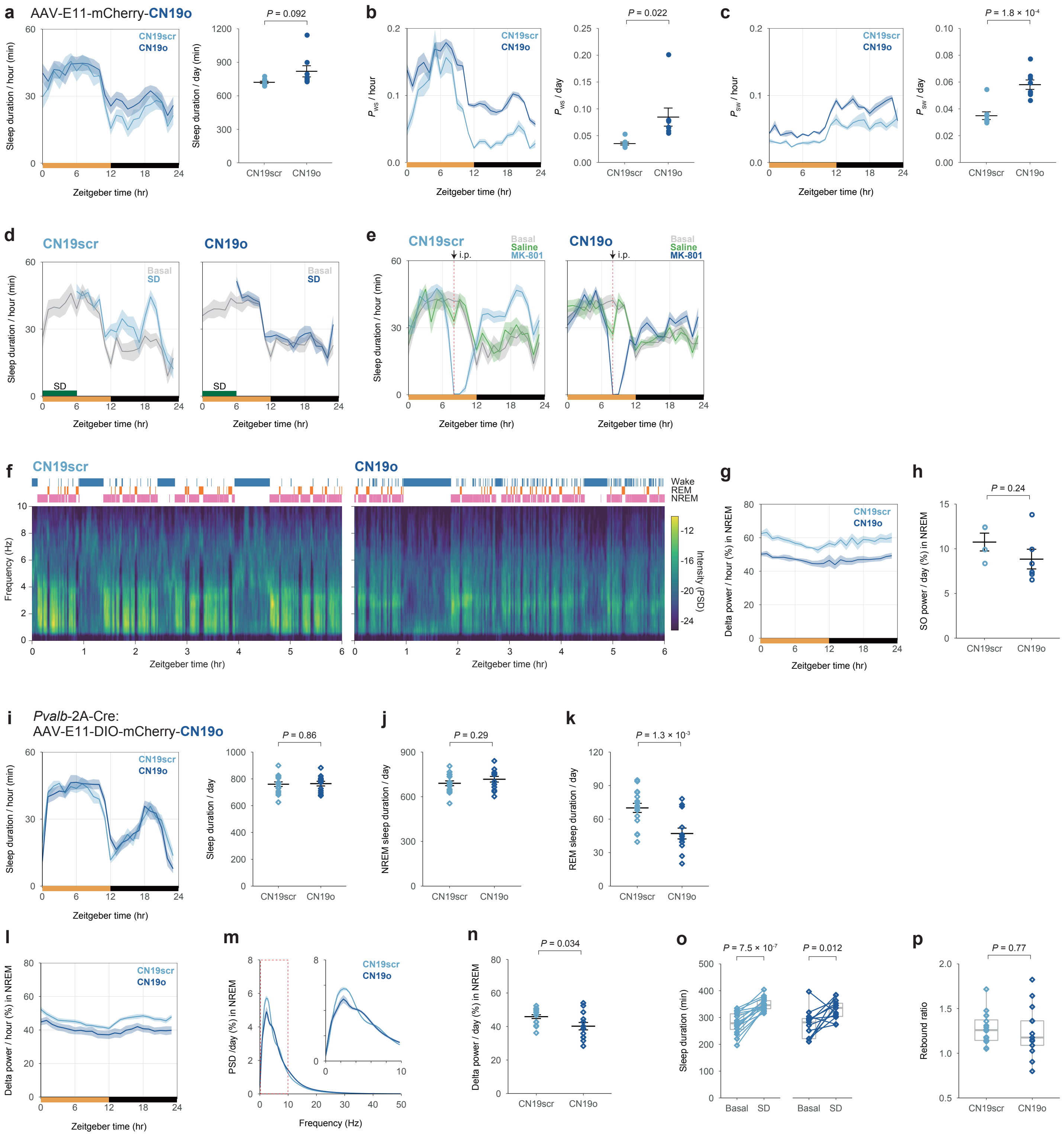

Supplementary Fig. 9

Supplementary Fig. 9: Inhibition of CaMKII kinase activity in E11 neurons affects sleep quality.

(a–c) Daily sleep duration **(a)**, P_{WS} **(b)**, and P_{SW} **(c)** in E11-CN19scr and E11-CN19o mice (*n* = 8 for each group). The groups were compared using two-sided Welch's *t*-test.

(d, e) Hourly sleep duration over 24 hours in SD **(d)** and MK-801 administration **(e)** experiments. Each mouse received i.p. injection in turn: saline or 2 mg/kg MK-801 injection at ZT8, with at least 2 days between injections **(e)**. Mice were allowed to behave freely for 3 days prior to SD or the first injection, and the averaged value of sleep parameters is shown as Basal.

(f) Representative sleep-wake features of E11-CN19scr (left) and E11-CN19o (right) mice are shown as hypnograms (top) and heat maps of EEG spectra (bottom).

(g) Hourly normalized delta power (0.5–4 Hz) during NREM sleep in E11-CN19scr (*n* = 4) and E11-CN19o (*n* = 6) mice.

(h) Normalized slow-oscillation (SO) power (0.5–1 Hz) during NREM sleep. The groups were compared using two-sided Welch's *t*-test.

(i–k) Daily sleep duration **(i)**, NREM sleep duration **(j)**, and REM sleep duration **(k)** in *Pvalb*-2A-Cre:E11-DIO-CN19scr (*n* = 16) and *Pvalb*-2A-Cre:E11-DIO-CN19o (*n* = 13) mice. The groups were compared using two-sided Welch's *t*-test.

(l) Hourly normalized delta power (0.5–4 Hz) during NREM sleep.

(m) Power spectral density (PSD) of the EEG during NREM sleep. The extracted diagram in the 0–10 Hz range is also shown in the top-right corner of the panel.

(n) Daily normalized delta power (0.5–4 Hz) during NREM sleep. The groups were compared using two-sided Welch's *t*-test.

(o) Sleep duration on Basal and SD during ZT12–24. Two-sided Welch's *t*-test was performed between Basal and SD within the groups.

(p) Sleep rebound ratio (SD / Basal during ZT12-24). The groups were compared using two-sided Welch's *t*-test.

Line and interval plots show mean ± SEM. In box plot, boxes show the median, 25th and 75th percentiles, and whiskers show minima to maxima excluding outliers. Source data are provided as a Source Data file.

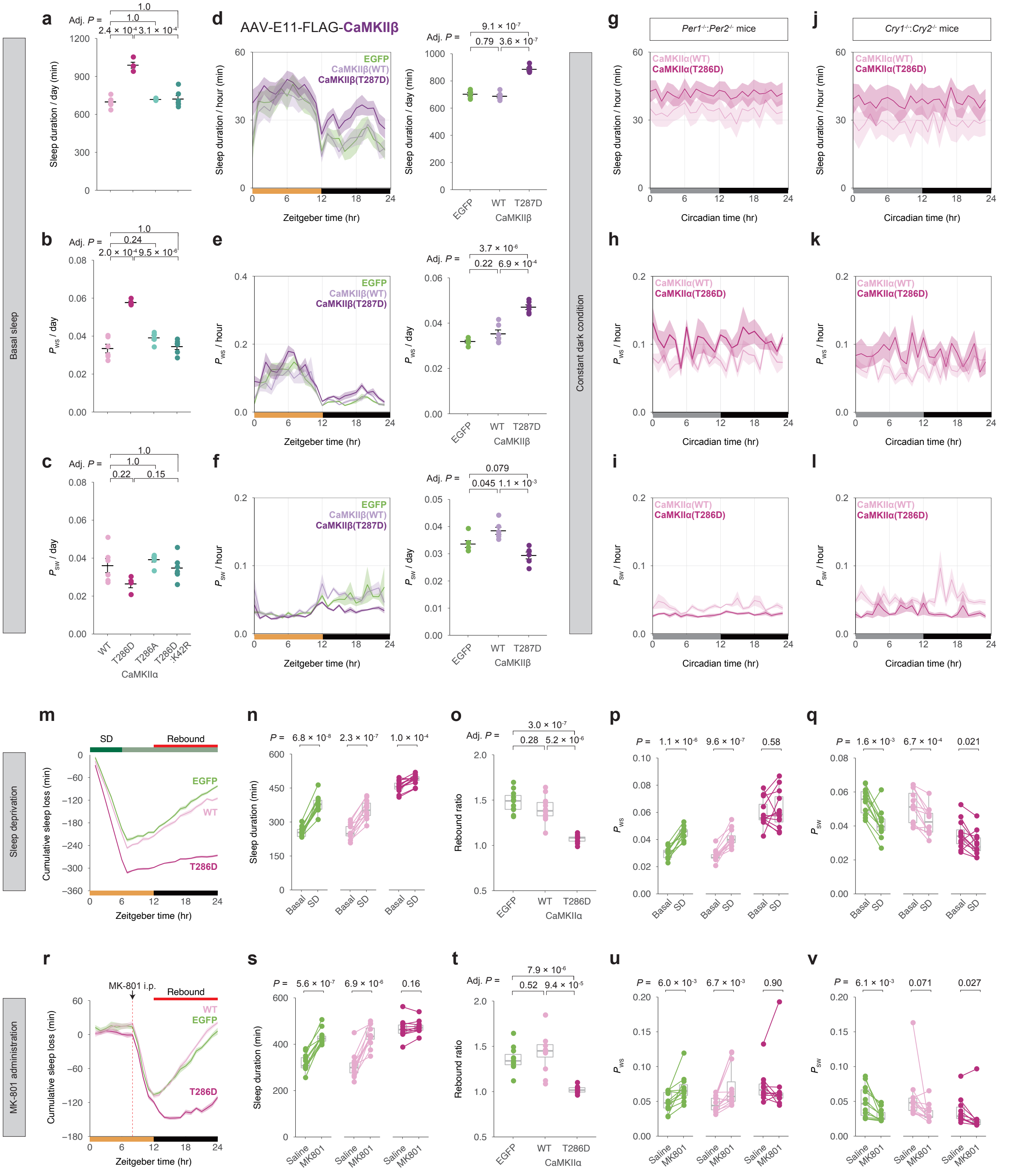

Supplementary Fig. 10

Supplementary Fig. 10: Elevation of CaMKIIα or β kinase activity in E11 neurons increases daily sleep amount and consolidates sleep state.

(a–c) Daily sleep duration **(a)**, P_{WS} **(b)**, and P_{SW} **(c)** in E11-CaMKIIα mutant mice (*n* = 6 for each group, except for T286D (*n* = 4)). Two-sided Welch's *t*-test with Bonferroni correction was used in comparison between the groups.

(d–f) Daily sleep duration **(d)**, P_{WS} **(e)**, and P_{SW} **(f)** in E11-EGFP, E11-CaMKIIβ (WT), and E11-CaMKIIβ (T287D) mice (*n* = 6 for each group). The groups were compared using two-sided Welch's *t*-test with the Bonferroni correction.

(g–i) Hourly sleep duration **(g)**, P_{WS} **(h)**, and P_{SW} **(i)** over 24 hours in the *Per* dKO mice expressing E11-CaMKIIα (WT) (*n* = 7) or E11-CaMKIIα (T286D) (*n* = 6).

(j–l) Hourly sleep duration **(j)**, P_{WS} **(k)**, and P_{SW} **(l)** over 24 hours in the *Cry* dKO mice expressing E11-CaMKIIα (WT) (*n* = 6) or E11-CaMKIIα (T286D) (*n* = 5).

(m) Cumulative sleep loss (SD – Basal) in E11-EGFP (*n* = 11), E11-CaMKIIα (WT) (*n* = 12), and E11-CaMKIIα (T286D) (*n* = 12) mice over 24 hours. Mice were allowed to behave freely for three days prior to SD, and the averaged value of sleep parameters is shown as Basal.

(r) Cumulative sleep loss (MK-801 – Saline) in E11-EGFP (*n* = 11), E11-CaMKIIα (WT) (*n* = 12), and E11-CaMKIIα (T286D) (*n* = 10) mice over 24 hours. Each mouse received i.p. injection in turn: saline or 2 mg/kg MK-801 injection at ZT8, allowing at least 2 days between injections.

(n, p, q, s, u, v) Sleep duration **(n, s)**, P_{WS} **(p, u)**, and P_{SW} **(q, v)** in SD **(n, p, q)** or MK-801 administration **(s, u, v)** during ZT12–24. Two-sided Student's paired *t*-test was used for comparison within the groups.

(o, t) Sleep rebound ratio in SD **(o)** or MK-801 administration **(t)**. The groups were compared using two-sided Welch's *t*-test with the Bonferroni correction.

Line and interval plots show mean ± SEM. In box plot, boxes show the median, 25th and 75th percentiles, and whiskers show minima to maxima excluding outliers. Source data are provided as a Source Data file.

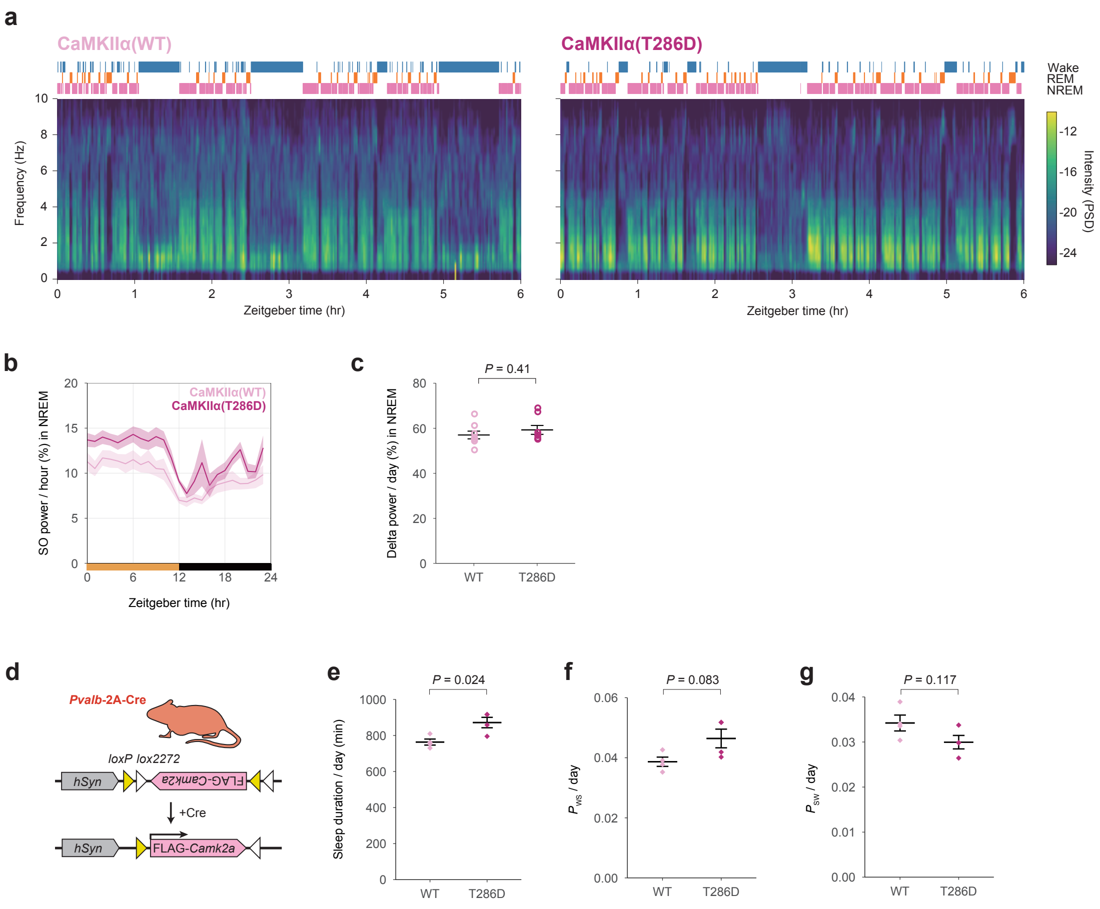

Supplementary Fig. 11

Supplementary Fig. 11: Elevation of CaMKIIα kinase activity in E11 neurons facilitates NREM sleep quality.

(a) Representative sleep-wake features of E11-CaMKIIα (WT) (left) and E11-CaMKIIα (T286D) (right) mice are shown as hypnograms (top) and heat maps of EEG spectra (bottom).

(b) Hourly normalized slow-oscillation (SO) power (0.5–1Hz) during NREM sleep in E11-CaMKIIα (WT) and E11-CaMKIIα (T286D) mice (n = 8 for each group).

(c) Daily normalized delta (0.5–4 Hz) power during NREM sleep. The groups were compared using two-sided Welch's t-test.

(d) Diagram of CaMKIIα expression in PV neurons using pan-neural *hSyn* promoter and *Pvalb*-2A-Cre mice.

(e–g) Daily sleep duration **(e)**, P_{WS} **(f)**, and P_{SW} **(g)** in *Pvalb*-2A-Cre:*hSyn*-DIO-CaMKIIα (WT) (n = 4) and *Pvalb*-2A-Cre:*hSyn*-DIO-CaMKIIα (T286D) (n = 3) mice. The groups were compared using two-sided Welch's t-test.

Line and interval plots show mean ± SEM. Source data are provided as a Source Data file.

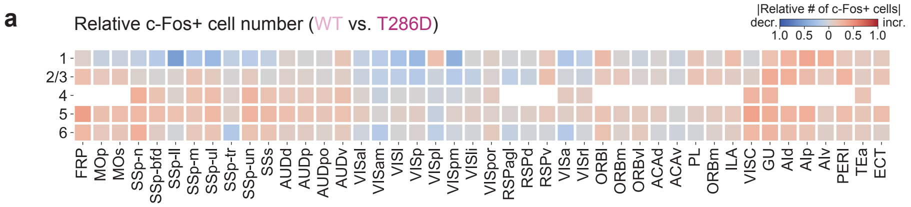

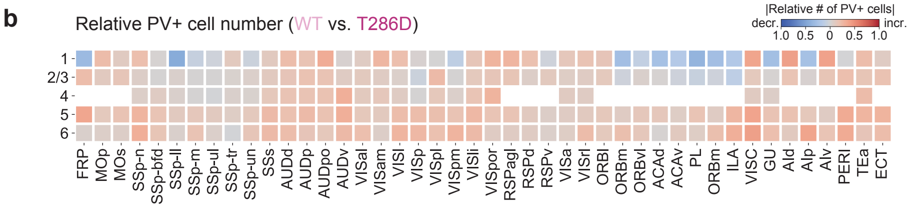

Supplementary Fig. 12

Supplementary Fig. 12: Analysis of cortical c-Fos+ or PV+ cells in elevation of CaMKIIα kinase activity in E11 neurons.

(a, b) The changes in c-Fos+ **(a)** and PV+ **(b)** cell number in the isocortex are represented as a heat map of relative cell number in each region. Red and blue colors represent a significant increase and decrease in the E11-CaMKIIα (T286D) brains compared to the E11-CaMKIIα (WT) brains, respectively.

The Allen Brain Atlas ontology is used to define brain region acronyms. Source data are provided as a Source Data file.

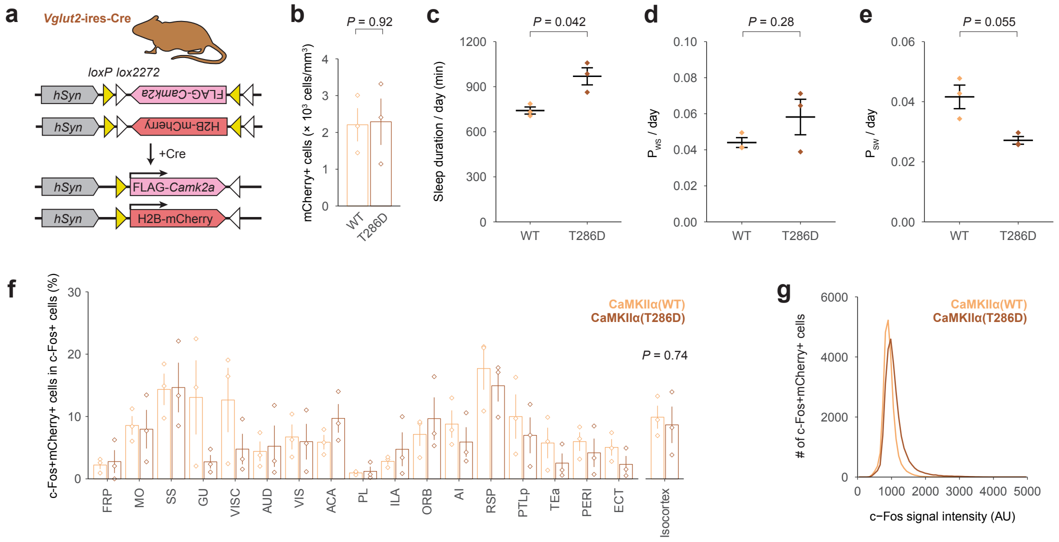

Supplementary Fig. 13

Supplementary Fig. 13: Analysis of cortical c-Fos+ cells in elevation of CaMKIIα kinase activity in excitatory neurons.

(a) Diagram of CaMKIIα and H2B-mCherry expression in excitatory neurons using *hSyn* promoter and *Vglut2*-ires-Cre mice.

(b) mCherry+ cell density in the isocortex in *Vglut2*-ires-Cre:*hSyn*-DIO-CaMKIIα (WT) and *Vglut2*-ires-Cre:*hSyn*-DIO-CaMKIIα (T286D) mouse brains (n = 3 for each group). The groups were compared using two-sided Welch's t-test.

(c–e) Daily sleep duration **(c)**, P_{WS} **(d)**, and P_{SW} **(e)** in *Vglut2*-ires-Cre:*hSyn*-DIO-CaMKIIα (WT) and *Vglut2*-ires-Cre:*hSyn*-DIO-CaMKIIα (T286D) mice. The groups were compared using two-sided Welch's t-test.

(f) The rate of double-positive (c-Fos+mCherry+) cells in c-Fos+ cells in each cortical region (left) or entire isocortex (right). The groups were compared using two-sided Welch's t-test.

(g) The cortical distribution of c-Fos signal intensity per c-Fos+mCherry+ cell in each group.

Bar and interval plots show mean ± SEM. The Allen Brain Atlas ontology is used to define brain region acronyms. Source data are provided as a Source Data file.

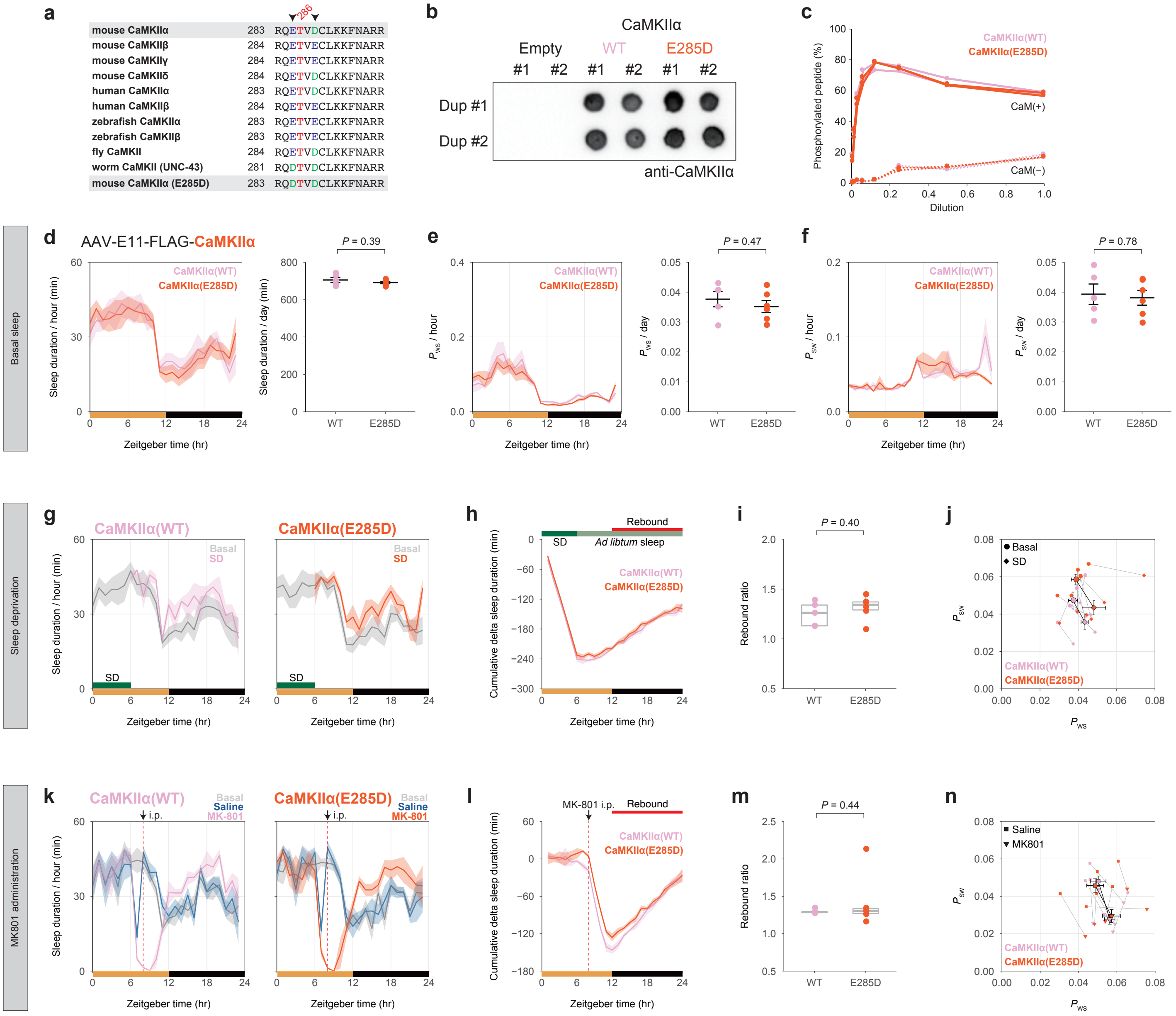

Supplementary Fig. 14

Supplementary Fig. 14: Watermark mutation E285D in CaMKIIα does not affect the kinase activity, sleep architecture, and sleep homeostasis.

(a) Sequence alignment of T286 (red) surrounding region of CaMKII family. The alignment was performed against the mouse (*M. musculus*) CaMKIIα/β/δ/γ, human (*H. sapiens*) CaMKIIα/β, zebrafish (*D. rerio*) CaMKIIα/β, fly (*D. melanogaster*) CaMKII, and worm (*C. elegans*) CaMKII analog (UNC-43). The residues of Glutamic acid (E) and aspartic acid (D) are colored in blue and green, respectively. The E285 and D288 positions of mouse CaMKIIα are indicated by black arrowheads.

(b) Dot blots of HEK293T cell lysates transfected CaMKIIα expression vectors with CaMKIIα antibody (n = 2 for each group). Vertical duplicates of each lysate were made.

(c) CaMKIIα kinase activity assay with CaMKII peptide substrate. The phosphorylated peptide was calculated as a percentage of the total peptide (n = 2 for each group).

(d–f) Daily sleep duration **(d)**, P_{WS} **(e)**, and P_{SW} **(f)** in E11-CaMKIIα (WT) (n = 5) and E11-CaMKIIα (E285D) (n = 6) mice. The groups were compared using two-sided Welch's *t*-test.

(g, h, k, l) Hourly sleep duration **(g, k)** and cumulative sleep loss **(h, l)** in E11-CaMKIIα (WT) (n = 5) and E11-CaMKIIα (E285D) (n = 6) mice in SD **(g, h)** or MK-801 administration **(k, l)**. Mice were allowed to behave freely for three days prior to SD or the saline injection, and the averaged value of sleep parameters is shown as Basal. Saline or 2 mg/kg MK-801 was injected at ZT8, allowing at least 2 days between injections.

(i, m) Sleep rebound ratio in SD **(i)** or MK-801 administration **(m)**. The groups were compared using two-sided Welch's *t*-test.

(j, n) P_{WS} and P_{SW} scatter diagrams in SD **(j)** or MK-801 administration **(n)**. Averaged values are represented by black-fringed dots, while individual mouse values are represented by other dots. Gray lines connect the dots of Basal and SD **(j)** or Saline and MK-801 **(n)** in each mouse.

Line and interval plots show mean ± SEM. Source data are provided as a Source Data file.

Supplementary Table 1: Summary of AAV applications and conditions.

Figure numbers	AAVs (AAV-PHP.eB)	Titer (vg/mouse)
Fig. 3j–l; Supplementary Fig. 5a–b	AAV-E11-H2B-mCherry-WPRE-SV40pA	5.0 × 10^11
Supplementary Fig. 5c–g	AAV-E11-TeLC-P2A-NLS-EGFP-WPRE-SV40pA	5.0 × 10^10
Fig. 3m–p; Supplementary Fig. 5h–m	AAV-E11-H2B-EGFP-WPRE-SV40pA	2.0 × 10^11
	AAV-E11-hM3Dq-mCherry-WPRE-hGHpA	
	AAV-E11-hM4Di-mCherry-WPRE-hGHpA	
Supplementary Fig. 6a–f	AAV-E22-H2B-EGFP-WPRE-SV40pA	2.0 × 10^11
	AAV-E22-hM3Dq-mCherry-WPRE-hGHpA	
	AAV-E22-hM4Di-mCherry-WPRE-hGHpA	
Fig. 4a–h; Supplementary Fig. 7a–g	AAV-E11-H2B-mCherry-WPRE-SV40pA	2.0 × 10^11
	AAV-E11-hM4Di-mCherry-WPRE-hGHpA	
Fig. 5a–p; Supplementary Fig. 9a–h	AAV-E11-mCherry-CN19scr-3'UTR(*Map2*)-WPRE-SV40pA	3.0 × 10^11
	AAV-E11-mCherry-CN19o-3'UTR(*Map2*)-WPRE-SV40pA	
Fig. 6a–d	AAV-E11-mCherry-CN19scr-3'UTR(*Map2*)-WPRE-SV40pA	5.0 × 10^11
	AAV-E11-mCherry-CN19o-3'UTR(*Map2*)-WPRE-SV40pA	
Fig. 6e–h; Supplementary Fig. 9i–n	AAV-E11-DIO-mCherry-CN19scr-3'UTR(*Map2*)-WPRE-SV40pA	1.5 × 10^12
	AAV-E11-DIO-mCherry-CN19o-3'UTR(*Map2*)-WPRE-SV40pA	
Fig. 7b–d; Supplementary Fig. 10m–v	AAV-E11-H2B-EGFP-WPRE-SV40pA	1.0 × 10^11
	AAV-E11-FLAG-CaMKIIα(WT)-3'UTR(*Camk2a*)-WPRE-SV40pA	
	AAV-E11-FLAG-CaMKIIα(T286D)-3'UTR(*Camk2a*)-WPRE-SV40pA	
Supplementary Fig. 10a–c	AAV-E11-FLAG-CaMKIIα(WT)-3'UTR(*Camk2a*)-WPRE-SV40pA	1.0 × 10^11
	AAV-E11-FLAG-CaMKIIα(T286D)-3'UTR(*Camk2a*)-WPRE-SV40pA	
	AAV-E11-FLAG-CaMKIIα(T286A)-3'UTR(*Camk2a*)-WPRE-SV40pA	
	AAV-E11-FLAG-CaMKIIα(T286D:K42R)-3'UTR(*Camk2a*)-WPRE-SV40pA	
Supplementary Fig. 10d–f	AAV-E11-H2B-EGFP-WPRE-SV40pA	1.0 × 10^11
	AAV-E11-FLAG-CaMKIIβ(WT)-3'UTR(*Camk2a*)-WPRE-SV40pA	
	AAV-E11-FLAG-CaMKIIβ(T287D)-3'UTR(*Camk2a*)-WPRE-SV40pA	
Fig. 7e–h; Supplementary Fig. 10g–l	AAV-E11-FLAG-CaMKIIα(WT)-3'UTR(*Camk2a*)-WPRE-SV40pA	1.0 × 10^11
	AAV-E11-FLAG-CaMKIIα(T286D)-3'UTR(*Camk2a*)-WPRE-SV40pA	
Fig. 7i–m; Supplementary Fig. 11a–c	AAV-E11-FLAG-CaMKIIα(WT)-3'UTR(*Camk2a*)-WPRE-SV40pA	1.0 × 10^11
	AAV-E11-FLAG-CaMKIIα(T286D)-3'UTR(*Camk2a*)-WPRE-SV40pA	
Fig. 7n–q	AAV-E11-DIO-FLAG-CaMKIIα(WT)-3'UTR(*Camk2a*)-WPRE-SV40pA	5.0 × 10^11
	AAV-E11-DIO-FLAG-CaMKIIα(T286D)-3'UTR(*Camk2a*)-WPRE-SV40pA	
Supplementary Fig. 11d–g	AAV-hSyn-DIO-FLAG-CaMKIIα(WT)-3'UTR(*Camk2a*)-WPRE-SV40pA	2.0 × 10^11
	AAV-hSyn-DIO-FLAG-CaMKIIα(T286D)-3'UTR(*Camk2a*)-WPRE-SV40pA	
Fig. 8a–e; Supplementary Fig. 12a–b	AAV-E11-FLAG-CaMKIIα(WT)-3'UTR(*Camk2a*)-WPRE-SV40pA	1.0 × 10^11
	AAV-E11-FLAG-CaMKIIα(T286D)-3'UTR(*Camk2a*)-WPRE-SV40pA	
Supplementary Fig. 13a–g	AAV-hSyn-DIO-FLAG-CaMKIIα(WT)-3'UTR(*Camk2a*)-WPRE-SV40pA; AAV-hSyn-DIO-H2BmCherry-WPRE-SV40pA	2.0 × 10^11; 2.0 × 10^11
	AAV-hSyn-DIO-FLAG-CaMKIIα(T286D)-3'UTR(*Camk2a*)-WPRE-SV40pA; AAV-hSyn-DIO-H2BmCherry-WPRE-SV40pA	
Fig. 8f–g; Supplementary Fig. 14d–n	AAV-E11-FLAG-CaMKIIα(WT)-3'UTR(*Camk2a*)-WPRE-SV40pA	5.0 × 10^11
	AAV-E11-FLAG-CaMKIIα(E285D)-3'UTR(*Camk2a*)-WPRE-SV40pA	
