# Supplementary material for: Cortical parvalbumin neurons are responsible for homeostatic sleep rebound through CaMKII activation
Source: Nat Commun. 2024 Jul 18;15:6054. doi: 10.1038/s41467-024-50168-5 (PMC11258272; doi:10.1038/s41467-024-50168-5)
Supplement: Supplementary file 3 — Reporting Summary [file 41467_2024_50168_MOESM3_ESM.pdf]

nature portfolio

Corresponding author(s): Hiroki R Ueda

Last updated by author(s): Jun 24, 2024

Reporting Summary

Nature Portfolio wishes to improve the reproducibility of the work that we publish. This form provides structure for consistency and transparency in reporting. For further information on Nature Portfolio policies, see our Editorial Policies and the Editorial Policy Checklist.

Statistics

For all statistical analyses, confirm that the following items are present in the figure legend, table legend, main text, or Methods section.

n/a	Confirmed	
☐	☒	The exact sample size (*n*) for each experimental group/condition, given as a discrete number and unit of measurement
☐	☒	A statement on whether measurements were taken from distinct samples or whether the same sample was measured repeatedly
☐	☒	The statistical test(s) used AND whether they are one- or two-sided<br>*Only common tests should be described solely by name; describe more complex techniques in the Methods section.*
☒	☐	A description of all covariates tested
☐	☒	A description of any assumptions or corrections, such as tests of normality and adjustment for multiple comparisons
☐	☒	A full description of the statistical parameters including central tendency (e.g. means) or other basic estimates (e.g. regression coefficient) AND variation (e.g. standard deviation) or associated estimates of uncertainty (e.g. confidence intervals)
☐	☒	For null hypothesis testing, the test statistic (e.g. F, t, r) with confidence intervals, effect sizes, degrees of freedom and P value noted<br>*Give P values as exact values whenever suitable.*
☒	☐	For Bayesian analysis, information on the choice of priors and Markov chain Monte Carlo settings
☒	☐	For hierarchical and complex designs, identification of the appropriate level for tests and full reporting of outcomes
☒	☐	Estimates of effect sizes (e.g. Cohen's *d*, Pearson's *r*), indicating how they were calculated

Our web collection on statistics for biologists contains articles on many of the points above.

Software and code

Policy information about availability of computer code

Data collection	LabVIEW (version 2016) for microscope operation. VitalRecorder 1.15.1.1812 (KISSEI COMTEC) for EEG/EMG data collection.
Data analysis	Python 3.8.2, R version 3.6.1, Microsoft Excel for Mac version 16.49, ilastik version 1.3.1, ANTs version 2.3.0, CUBIC-Cloud (CUBICStars), Xcalibur 4.4 (Thermo Fisher Scientific), Proteome Discoverer version 2.5 (Thermo Fisher Scientific), and LabChip EZ reader 3.0.265.0 SP2 (Perkin Elmer) for data analyses and figure preparation.

For manuscripts utilizing custom algorithms or software that are central to the research but not yet described in published literature, software must be made available to editors and reviewers. We strongly encourage code deposition in a community repository (e.g. GitHub). See the Nature Portfolio guidelines for submitting code & software for further information.

Data

Policy information about availability of data

All manuscripts must include a data availability statement. This statement should provide the following information, where applicable:

- Accession codes, unique identifiers, or web links for publicly available datasets
- A description of any restrictions on data availability
- For clinical datasets or third party data, please ensure that the statement adheres to our policy

Protein mass spectrometry raw data have been deposited in the MassIVE dataset, a member of ProteomeXchange consortium (MSV000095087). Due to the large

size of raw data, other data used in this study is available from the corresponding author upon request. Source data generated in this study are provided in the Source Data file. Source data are provided with this paper.

Research involving human participants, their data, or biological material

Policy information about studies with human participants or human data. See also policy information about sex, gender (identity/presentation), and sexual orientation and race, ethnicity and racism.

Reporting on sex and gender	NA
Reporting on race, ethnicity, or other socially relevant groupings	NA
Population characteristics	NA
Recruitment	NA
Ethics oversight	NA

Note that full information on the approval of the study protocol must also be provided in the manuscript.

Field-specific reporting

Please select the one below that is the best fit for your research. If you are not sure, read the appropriate sections before making your selection.

☒ Life sciences ☐ Behavioural & social sciences ☐ Ecological, evolutionary & environmental sciences

For a reference copy of the document with all sections, see nature.com/documents/nr-reporting-summary-flat.pdf

Life sciences study design

All studies must disclose on these points even when the disclosure is negative.

Sample size	No statistical method was used to predetermine the sample size. The sample sizes were determined based on previous experiences and reports.
Data exclusions	Individuals with abnormal measurement signals or weakened individuals were excluded from the sleep data analyses because of their difficulties in accurate sleep phenotyping. For imaging and biochemical analysis, no data was excluded.
Replication	Sleep and biochemical experiments were repeated at least two times with the independent sets of the animals or independently prepared cell lysates. Mouse brains for imaging analysis were prepared in 2 or more separate batches. All attempts at replication were successful.
Randomization	The mice used in each experiment were randomly chosen from colonies.
Blinding	All sleep measurement data was automatically analyzed, and EEG/EMG data was blinded for manual check of sleep stage annotations. Imaging analysis was conducted using automated workflow.

Reporting for specific materials, systems and methods

We require information from authors about some types of materials, experimental systems and methods used in many studies. Here, indicate whether each material, system or method listed is relevant to your study. If you are not sure if a list item applies to your research, read the appropriate section before selecting a response.

Materials & experimental systems

n/a	Involved in the study	
☐	☒	Antibodies
☐	☒	Eukaryotic cell lines
☒	☐	Palaeontology and archaeology
☐	☒	Animals and other organisms
☒	☐	Clinical data
☒	☐	Dual use research of concern
☒	☐	Plants

Methods

n/a	Involved in the study	
☒	☐	ChIP-seq
☒	☐	Flow cytometry
☒	☐	MRI-based neuroimaging

Antibodies

Antibodies used	Antigen, Vendor, Catalog #, Host species, dilution<br>PV, Swant, #PV235, Mouse, 1:50 or 1:20<br>c-Fos, CST, #2250S, Rabbit, 1:26<br>CaMKIIα, StressMarq, SMC-124D, Mouse, 1:3000<br>Mouse IgG1, Jackson ImmunoResearch, #115-167-185, Goat, 1:150 or 1:80<br>Rabbit IgG, Jackson ImmunoResearch, #111-607-008, Goat, 1:80
Validation	Antibody validation has been done by the antibody vendors. Information on antibody validation is available on the vendors' websites for PV (https://webshop.swant.com/pv235-parvalbumin.html), c-Fos (https://www.cellsignal.com/products/primary-antibodies/c-fos-9f6-rabbit-mab/2250), and CaMKIIα (https://www.stressmarq.com/products/antibodies/monoclonal-antibodies/camkii-alpha-specific-antibody-smc-124/). In dot blots of HEK 293T cell samples, the CaMKIIα antibody specifically showed a strong signal in HEK 293T cell samples transfected with the mouse Camk2a expression vector.

Eukaryotic cell lines

Policy information about cell lines and Sex and Gender in Research

Cell line source(s)	The HEK 293T cell line (CRL-3216, ATCC) and the AAVpro 293T cell line (632273, Takara Bio) were obtained from ATCC and Takara Bio, respectively.
Authentication	None of the cell lines used were authenticated.
Mycoplasma contamination	All cell lines tested negative for mycoplasma.
Commonly misidentified lines<br>(See ICLAC register)	NA

Animals and other research organisms

Policy information about studies involving animals; ARRIVE guidelines recommended for reporting animal research, and Sex and Gender in Research

Laboratory animals	3-12-week-old male C57BL/6NJcl mice (CLEA Japan Inc.) for sleep, imaging, and biochemical experiments.<br>The following mice were produced using ES cells: Pvalb-2A-Cre mice; Vglut2-ires-Cre mice; Cry1 -/-:Cry2 -/- mice; Per1 -/-:Per2 -/- mice. The Animal Care and Use Committee of the Graduate School of Medicine in the University of Tokyo or the Institutional Animal Care and Use Committee of RIKEN BDR approved all animal care and experimental procedures.
Wild animals	No wild animal was used.
Reporting on sex	Male mice were used in this study. Female mice were not used because they potentially show the estrous-cycle-dependent sleep changes.
Field-collected samples	No Field-collected samples were used.
Ethics oversight	All experimental procedures and housing conditions were approved by the Institutional Animal Care and Use Committee of the University of Tokyo and RIKEN Center for Biosystems Dynamics Research.

Note that full information on the approval of the study protocol must also be provided in the manuscript.
