# Supplementary figures and images for: Cortical parvalbumin neurons are responsible for homeostatic sleep rebound through CaMKII activation
Source: Nat Commun. 2024 Jul 18;15:6054. doi: 10.1038/s41467-024-50168-5 (PMC11258272; doi:10.1038/s41467-024-50168-5)

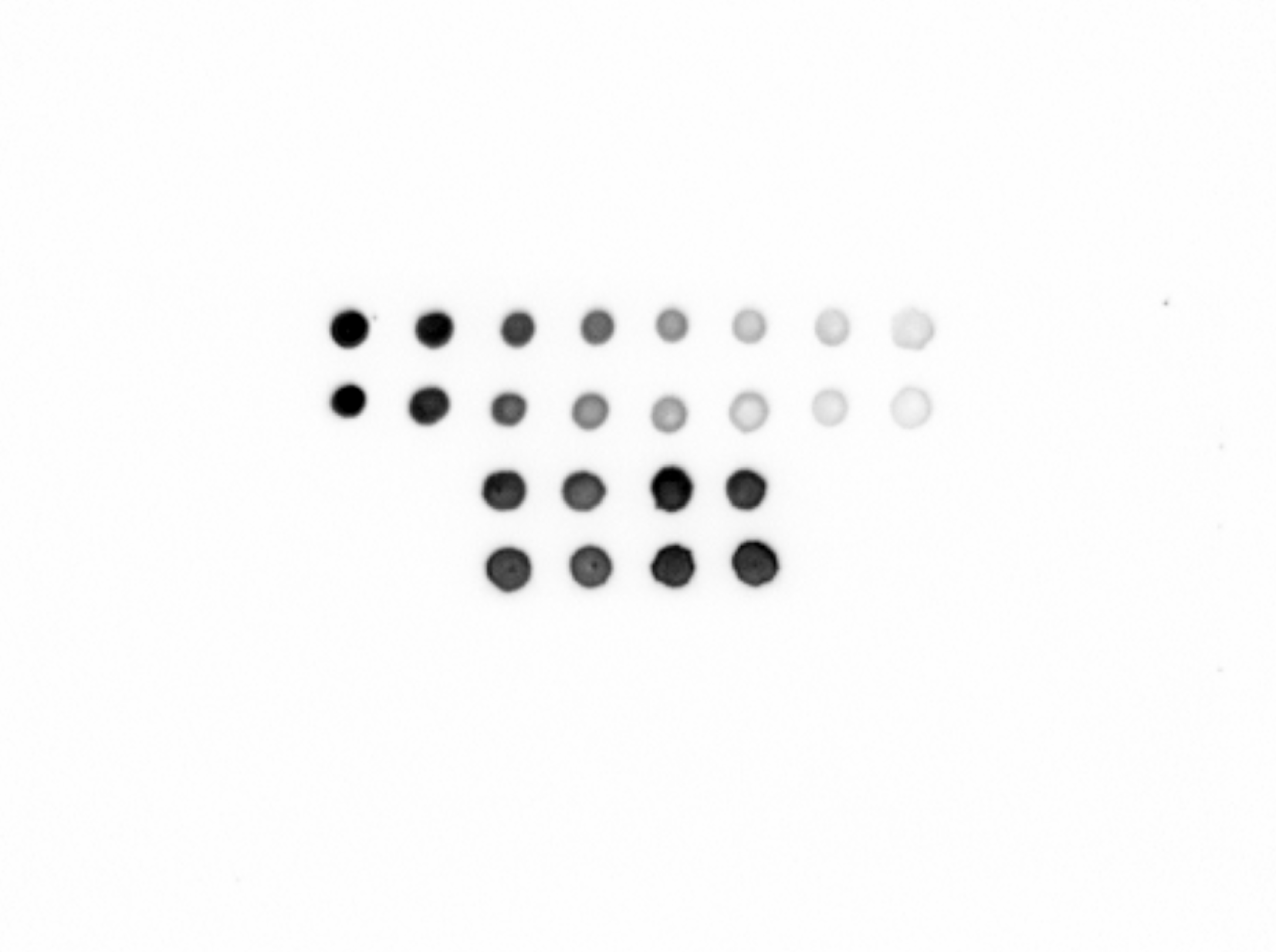

Supplement: Supplementary file 4 — Source Data [file 41467_2024_50168_MOESM4_ESM.zip › Source Data/uncropped_image_supp_fig14b.tif]
